# 8-(4-Nitro­benz­yloxy)quinoline

**DOI:** 10.1107/S1600536809033212

**Published:** 2009-08-29

**Authors:** Yuan Zhang, Yong Hua Li

**Affiliations:** a Ordered Matter Science Research Center, College of Chemistry and Chemical Engineering, Southeast University, Nanjing 211189, People’s Republic of China

## Abstract

In the title compound, C_16_H_12_N_2_O_3_, the planar quinoline ring system [maximum deviation = 0.025 (3) Å] is oriented at a dihedral angle of 61.76 (7)° with respect to the benzene ring. In the crystal structure, inter­molecular C—H⋯O inter­actions link the mol­ecules into chains parallel to the *b* axis. π–π contacts between the quinoline rings [centroid–centroid distance = 3.623 (1) Å] may further stabilize the structure.

## Related literature

For related structures, see: Fu & Zhao (2007 ▶); Li & Chen (2008 ▶); Zhao (2008 ▶). For bond-length data, see: Allen *et al.* (1987 ▶).
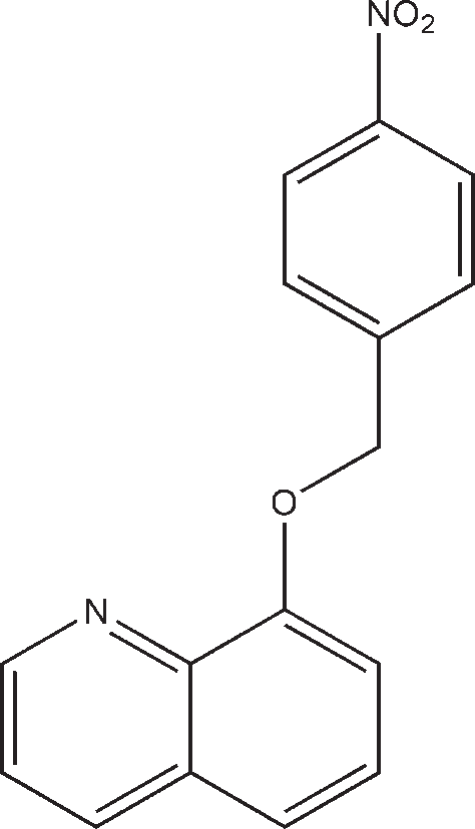


## Experimental

### 

#### Crystal data

C_16_H_12_N_2_O_3_
*M* *_r_* = 280.28
Monoclinic, 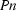

*a* = 4.176 (3) Å
*b* = 7.395 (3) Å
*c* = 21.513 (18) Å
β = 94.08 (3)°
*V* = 662.7 (8) Å^3^
*Z* = 2
Mo *K*α radiation
μ = 0.10 mm^−1^
*T* = 294 K
0.20 × 0.20 × 0.20 mm

#### Data collection

Rigaku SCXmini diffractometer
Absorption correction: multi-scan (*CrystalClear*, Rigaku, 2005 ▶)*T*_min_ = 0.789, *T*_max_ = 0.980
5732 measured reflections
2566 independent reflections
2134 reflections with *I* > 2σ(*I*)
*R*_int_ = 0.028

#### Refinement

*R*[*F* ^2^ > 2σ(*F* ^2^)] = 0.042
*wR*(*F* ^2^) = 0.086
*S* = 1.01
2566 reflections
191 parameters
H-atom parameters constrained
Δρ_max_ = 0.17 e Å^−3^
Δρ_min_ = −0.16 e Å^−3^

### 

Data collection: *CrystalClear* (Rigaku, 2005 ▶); cell refinement: *CrystalClear*; data reduction: *CrystalClear*; program(s) used to solve structure: *SHELXS97* (Sheldrick, 2008 ▶); program(s) used to refine structure: *SHELXL97* (Sheldrick, 2008 ▶); molecular graphics: *SHELXTL/PC* (Sheldrick, 2008 ▶) and *PLATON* (Spek, 2009 ▶); software used to prepare material for publication: *SHELXTL/PC* and *PLATON*.

## Supplementary Material

Crystal structure: contains datablocks I, global. DOI: 10.1107/S1600536809033212/hk2759sup1.cif

Structure factors: contains datablocks I. DOI: 10.1107/S1600536809033212/hk2759Isup2.hkl

Additional supplementary materials:  crystallographic information; 3D view; checkCIF report

## Figures and Tables

**Table 1:** Hydrogen-bond geometry (Å, °)

*D*—H⋯*A*	*D*—H	H⋯*A*	*D*⋯*A*	*D*—H⋯*A*
C10—H10*A*⋯O2^i^	0.97	2.60	3.538 (3)	164

Symmetry code: (i) 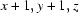.

**Fig. 1.**
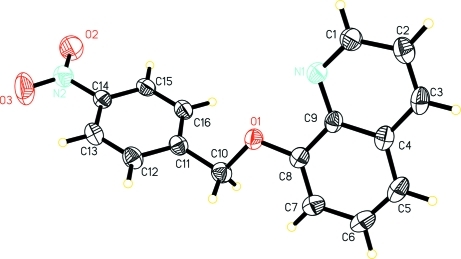
The molecular structure of the title molecule with the atom-numbering scheme. Displacement ellipsoids are drawn at the 30% probability level.

**Fig. 2.**
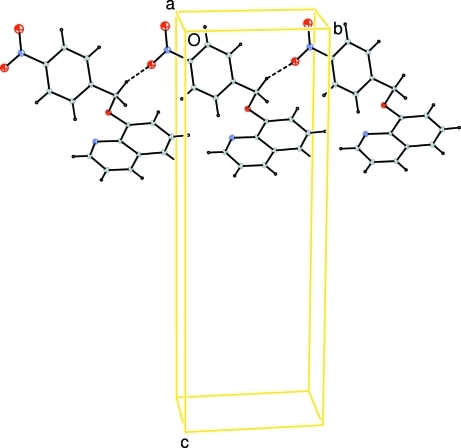
A partial packing diagram of the title compound. Hydrogen bonds are shown as dashed lines.

## supplementary crystallographic information

### Comment

Recently, we have reported the syntheses and crystal structures of some benzonitrile compounds (Fu & Zhao, 2007; Li & Chen, 2008; Zhao, 2008). As an extension of our work on the structural characterizations of benzonitrile derivatives, we report herein the synthesis and crystal structure of the title compound.

In the molecule of the title compound, (Fig. 1), the bond lengths (Allen *et al.*,  1987) and angles are within normal ranges. The quinoline ring system is planar with a maximum deviation of 0.025 (3) Å for atom C6, and it is oriented with respect to the benzene ring at a dihedral angle of 61.76 (7)°.

In the crystal structure, intermolecular C-H···O interactions (Table 1) link the molecules into chains paralel to the b axis (Fig. 2), in which they may be effective in the stabilization of the structure. The π–π contact between the quinoline rings, Cg1—Cg2^i^ [symmetry code: (i) 1 + x, y, z, where Cg1 and Cg2 are centroids of the rings (N1/C1-C4/C9) and (C4-C9), respectively] may further stabilize the structure, with centroid-centroid distance of 3.623 (1) Å.

### Experimental

For the preparation of the title compound, quinolin-8-ol (1 g, 0.0069 mol) was added to a solution of sodium hydroxide (0.276 g, 0.0069 mol) in methanol (15 ml) and stirred for 3 h. Then, 1-(bromomethyl)-4-nitrobenzene (1.5318 g, 0.0069 mol) was added. The mixture was stirred at room temperature for 2 d. The title compound was isolated using column chromatography (petroleum ether: ethyl acetate, 1:1). Crystals suitable for X-ray analysis were obtained from slow evaporation of an ethyl acetate and tetrahydrofuran solution.

### Refinement

H atoms were positioned geometrically with C-H = 0.93 and 0.97 Å for aromatic and methylene H atoms, respectively, and constrained to ride on their parent atoms, with U_iso_(H) = 1.2U_eq_(C). The absolute structure could not be determined reliably, and 1267 Friedel pairs were averaged before the last cycle of refinement.

### Crystal data

**Table:**

C_16_H_12_N_2_O_3_	*F*(000) = 292
*M**_r_* = 280.28	*D*_x_ = 1.405 Mg m^−^^3^
Monoclinic, *P**n*	Mo *K*α radiation, λ = 0.71073 Å
Hall symbol: P -2yac	Cell parameters from 1688 reflections
*a* = 4.176 (3) Å	θ = 2.8–27.5°
*b* = 7.395 (3) Å	µ = 0.10 mm^−^^1^
*c* = 21.513 (18) Å	*T* = 294 K
β = 94.08 (3)°	Block, pale yellow
*V* = 662.7 (8) Å^3^	0.20 × 0.20 × 0.20 mm
*Z* = 2	

### Data collection

**Table:**

Rigaku SCXmini diffractometer	2566 independent reflections
Radiation source: fine-focus sealed tube	2134 reflections with *I* > 2σ(*I*)
graphite	*R*_int_ = 0.028
CCD_Profile_fitting scans	θ_max_ = 26.0°, θ_min_ = 2.9°
Absorption correction: multi-scan (*CrystalClear*, Rigaku, 2005)	*h* = −5→5
*T*_min_ = 0.789, *T*_max_ = 0.980	*k* = −9→9
5732 measured reflections	*l* = −26→26

### Refinement

**Table:**

Refinement on *F*^2^	Hydrogen site location: inferred from neighbouring sites
Least-squares matrix: full	H-atom parameters constrained
*R*[*F*^2^ > 2σ(*F*^2^)] = 0.042	*w* = 1/[σ^2^(*F*_o_^2^) + (0.0196*P*)^2^ + 0.15*P*] where *P* = (*F*_o_^2^ + 2*F*_c_^2^)/3
*wR*(*F*^2^) = 0.086	(Δ/σ)_max_ < 0.001
*S* = 1.01	Δρ_max_ = 0.17 e Å^−^^3^
2566 reflections	Δρ_min_ = −0.16 e Å^−^^3^
191 parameters	Extinction correction: *SHELXL97* (Sheldrick, 2008), Fc^*^=kFc[1+0.001xFc^2^λ^3^/sin(2θ)]^-1/4^
Primary atom site location: structure-invariant direct methods	Extinction coefficient: 0.036 (3)
Secondary atom site location: difference Fourier map	

### Special details

**Table:**

Geometry. All e.s.d.'s (except the e.s.d. in the dihedral angle between two l.s. planes) are estimated using the full covariance matrix. The cell e.s.d.'s are taken into account individually in the estimation of e.s.d.'s in distances, angles and torsion angles; correlations between e.s.d.'s in cell parameters are only used when they are defined by crystal symmetry. An approximate (isotropic) treatment of cell e.s.d.'s is used for estimating e.s.d.'s involving l.s. planes.
Refinement. Refinement of *F*^2^ against ALL reflections. The weighted *R*-factor *wR* and goodness of fit *S* are based on *F*^2^, conventional *R*-factors *R* are based on *F*, with *F* set to zero for negative *F*^2^. The threshold expression of *F*^2^ > σ(*F*^2^) is used only for calculating *R*-factors(gt) *etc*. and is not relevant to the choice of reflections for refinement. *R*-factors based on *F*^2^ are statistically about twice as large as those based on *F*, and *R*- factors based on ALL data will be even larger.

### Fractional atomic coordinates and isotropic or equivalent isotropic displacement parameters (Å^2^)

**Table:**

	*x*	*y*	*z*	*U*_iso_*/*U*_eq_	
O1	0.8658 (4)	0.5082 (2)	0.23916 (8)	0.0539 (4)	
O2	0.1576 (6)	−0.2236 (3)	0.08570 (10)	0.1024 (8)	
O3	0.3470 (9)	−0.1172 (4)	0.00328 (11)	0.1334 (11)	
N1	1.2733 (5)	0.4283 (2)	0.33661 (9)	0.0531 (5)	
N2	0.2926 (6)	−0.1057 (3)	0.05809 (11)	0.0727 (7)	
C1	1.4711 (6)	0.3933 (4)	0.38541 (12)	0.0609 (7)	
H1A	1.5451	0.2752	0.3903	0.073*	
C2	1.5794 (6)	0.5201 (4)	0.43068 (12)	0.0654 (7)	
H2A	1.7160	0.4858	0.4647	0.078*	
C3	1.4798 (6)	0.6939 (4)	0.42365 (11)	0.0612 (7)	
H3A	1.5518	0.7809	0.4526	0.073*	
C4	1.2677 (5)	0.7427 (3)	0.37262 (11)	0.0525 (6)	
C5	1.1593 (7)	0.9231 (4)	0.36205 (14)	0.0649 (7)	
H5A	1.2225	1.0140	0.3902	0.078*	
C6	0.9646 (6)	0.9620 (4)	0.31112 (14)	0.0665 (8)	
H6A	0.8982	1.0807	0.3040	0.080*	
C7	0.8594 (6)	0.8260 (3)	0.26827 (12)	0.0584 (7)	
H7A	0.7238	0.8555	0.2336	0.070*	
C8	0.9567 (5)	0.6509 (3)	0.27772 (11)	0.0482 (6)	
C9	1.1702 (5)	0.6040 (3)	0.33024 (11)	0.0464 (5)	
C10	0.6534 (6)	0.5515 (3)	0.18613 (12)	0.0561 (6)	
H10A	0.7577	0.6328	0.1585	0.067*	
H10B	0.4614	0.6100	0.1992	0.067*	
C11	0.5679 (5)	0.3764 (3)	0.15321 (11)	0.0497 (6)	
C12	0.6351 (5)	0.3513 (4)	0.09144 (11)	0.0582 (6)	
H12A	0.7404	0.4417	0.0708	0.070*	
C13	0.5469 (6)	0.1932 (3)	0.06027 (12)	0.0598 (6)	
H13A	0.5918	0.1769	0.0189	0.072*	
C14	0.3921 (6)	0.0609 (3)	0.09139 (11)	0.0518 (6)	
C15	0.3210 (6)	0.0814 (3)	0.15294 (11)	0.0548 (6)	
H15A	0.2155	−0.0096	0.1733	0.066*	
C16	0.4104 (5)	0.2402 (3)	0.18354 (10)	0.0537 (6)	
H16A	0.3646	0.2560	0.2249	0.064*	

### Atomic displacement parameters (Å^2^)

**Table:**

	*U*^11^	*U*^22^	*U*^33^	*U*^12^	*U*^13^	*U*^23^
O1	0.0583 (10)	0.0516 (9)	0.0505 (9)	0.0015 (8)	−0.0043 (7)	−0.0077 (7)
O2	0.149 (2)	0.0699 (13)	0.0880 (17)	−0.0360 (14)	0.0046 (15)	−0.0007 (13)
O3	0.228 (3)	0.1087 (19)	0.0649 (14)	−0.057 (2)	0.0222 (17)	−0.0289 (14)
N1	0.0568 (12)	0.0473 (11)	0.0548 (11)	−0.0060 (9)	0.0017 (10)	−0.0030 (10)
N2	0.0905 (18)	0.0683 (16)	0.0572 (14)	−0.0059 (13)	−0.0099 (13)	−0.0036 (12)
C1	0.0636 (17)	0.0560 (15)	0.0618 (16)	−0.0086 (13)	−0.0033 (13)	0.0008 (13)
C2	0.0620 (17)	0.080 (2)	0.0534 (15)	−0.0124 (15)	−0.0033 (13)	−0.0022 (14)
C3	0.0614 (16)	0.0704 (19)	0.0524 (15)	−0.0220 (14)	0.0078 (12)	−0.0170 (13)
C4	0.0507 (13)	0.0567 (15)	0.0520 (13)	−0.0156 (12)	0.0157 (11)	−0.0109 (12)
C5	0.0697 (18)	0.0510 (15)	0.0759 (18)	−0.0157 (13)	0.0195 (15)	−0.0188 (14)
C6	0.0714 (19)	0.0420 (15)	0.088 (2)	−0.0021 (12)	0.0233 (16)	−0.0033 (14)
C7	0.0585 (15)	0.0511 (16)	0.0665 (16)	0.0000 (12)	0.0101 (12)	0.0025 (13)
C8	0.0479 (13)	0.0472 (14)	0.0510 (13)	−0.0057 (11)	0.0133 (10)	−0.0037 (11)
C9	0.0484 (14)	0.0470 (13)	0.0449 (12)	−0.0104 (10)	0.0103 (10)	−0.0073 (10)
C10	0.0511 (15)	0.0613 (16)	0.0549 (15)	0.0019 (12)	−0.0036 (12)	0.0012 (12)
C11	0.0415 (12)	0.0599 (16)	0.0467 (13)	0.0017 (11)	−0.0030 (10)	0.0009 (11)
C12	0.0556 (14)	0.0696 (16)	0.0495 (14)	−0.0104 (12)	0.0040 (11)	0.0031 (13)
C13	0.0619 (15)	0.0740 (18)	0.0432 (13)	−0.0028 (14)	0.0021 (11)	0.0003 (13)
C14	0.0559 (14)	0.0521 (14)	0.0464 (13)	0.0021 (11)	−0.0046 (11)	0.0017 (11)
C15	0.0614 (16)	0.0559 (15)	0.0470 (13)	0.0009 (12)	0.0041 (11)	0.0102 (11)
C16	0.0545 (14)	0.0630 (16)	0.0438 (12)	0.0039 (11)	0.0043 (10)	0.0038 (11)

### Geometric parameters (Å, °)

**Table:**

O1—C8	1.379 (3)	C8—C7	1.367 (3)
O1—C10	1.431 (3)	C9—N1	1.372 (3)
N1—C1	1.315 (3)	C9—C4	1.413 (3)
N2—O2	1.216 (3)	C9—C8	1.431 (3)
N2—O3	1.220 (3)	C10—C11	1.507 (3)
N2—C14	1.470 (3)	C10—H10A	0.9700
C1—C2	1.403 (3)	C10—H10B	0.9700
C1—H1A	0.9300	C11—C16	1.391 (3)
C2—H2A	0.9300	C12—C11	1.390 (3)
C3—C2	1.356 (4)	C12—C13	1.385 (3)
C3—H3A	0.9300	C12—H12A	0.9300
C4—C3	1.408 (3)	C13—C14	1.373 (3)
C4—C5	1.422 (3)	C13—H13A	0.9300
C5—C6	1.348 (4)	C15—C14	1.386 (3)
C5—H5A	0.9300	C15—C16	1.385 (3)
C6—H6A	0.9300	C15—H15A	0.9300
C7—C6	1.413 (4)	C16—H16A	0.9300
C7—H7A	0.9300		
			
C8—O1—C10	115.90 (19)	C7—C8—C9	120.6 (2)
C1—N1—C9	116.2 (2)	N1—C9—C4	123.3 (2)
O2—N2—C14	119.2 (2)	N1—C9—C8	118.80 (18)
O3—N2—O2	123.2 (3)	C4—C9—C8	117.9 (2)
O3—N2—C14	117.7 (3)	O1—C10—C11	107.2 (2)
N1—C1—C2	125.2 (3)	O1—C10—H10A	110.3
N1—C1—H1A	117.4	O1—C10—H10B	110.3
C2—C1—H1A	117.4	C11—C10—H10A	110.3
C1—C2—H2A	120.8	C11—C10—H10B	110.3
C3—C2—C1	118.3 (3)	H10A—C10—H10B	108.5
C3—C2—H2A	120.8	C12—C11—C10	120.5 (2)
C2—C3—C4	120.1 (2)	C12—C11—C16	119.1 (2)
C2—C3—H3A	120.0	C16—C11—C10	120.4 (2)
C4—C3—H3A	120.0	C11—C12—H12A	119.6
C3—C4—C9	117.0 (2)	C13—C12—C11	120.7 (2)
C3—C4—C5	122.7 (2)	C13—C12—H12A	119.6
C9—C4—C5	120.3 (2)	C12—C13—H13A	120.5
C4—C5—H5A	120.1	C14—C13—C12	118.9 (2)
C6—C5—C4	119.8 (2)	C14—C13—H13A	120.5
C6—C5—H5A	120.1	C13—C14—N2	119.1 (2)
C5—C6—C7	121.3 (3)	C13—C14—C15	121.9 (2)
C5—C6—H6A	119.3	C15—C14—N2	119.0 (2)
C7—C6—H6A	119.3	C14—C15—H15A	120.7
C6—C7—H7A	120.0	C16—C15—C14	118.6 (2)
C8—C7—C6	120.1 (2)	C16—C15—H15A	120.7
C8—C7—H7A	120.0	C11—C16—H16A	119.6
O1—C8—C9	114.75 (19)	C15—C16—C11	120.8 (2)
C7—C8—O1	124.7 (2)	C15—C16—H16A	119.6

### Hydrogen-bond geometry (Å, °)

**Table:**

*D*—H···*A*	*D*—H	H···*A*	*D*···*A*	*D*—H···*A*
C10—H10A···O2^i^	0.97	2.60	3.538 (3)	164

Symmetry codes: (i) *x*+1, *y*+1, *z*.
